# A pragmatic lifestyle intervention for overweight and obese women with gestational diabetes mellitus (PAIGE2): A parallel arm, multicenter randomized controlled trial study protocol

**DOI:** 10.3389/fcdhc.2023.1118509

**Published:** 2023-03-24

**Authors:** Emma McAuley, Olwen Fleck, Laura Cassidy, Bridie J. Kemp, Gina Cupples, Bronagh Kelly, Rachel M. Creighton, Una Graham, Helen Wallace, Chris C. Patterson, David R. McCance

**Affiliations:** ^1^ Regional Centre for Endocrinology and Diabetes, Royal Jubilee Maternity Centre, Belfast Health and Social Care Trust, Belfast, United Kingdom; ^2^ School of Nursing and Midwifery, Queen’s University Belfast, Belfast, United Kingdom; ^3^ Centre for Public Health, School of Medicine, Dentistry and Biomedical Sciences, Queen’s University Belfast, Belfast, United Kingdom

**Keywords:** gestational diabetes mellitus, lifestyle, overweight, obesity, pregnancy, randomized controlled trial

## Abstract

**Background:**

The global epidemic of type 2 diabetes (T2D) and obesity has been translated into pregnancy, with approximately 18% of women being diagnosed worldwide with Gestational Diabetes Mellitus (GDM). Whilst preventive strategies have proven effective in the non-pregnant context, attrition rates are high and there is an urgent need to develop a customized, pragmatic lifestyle intervention for women both during and after pregnancy. Diet and exercise modification, behavioral support, and Commercial Weight Management Organizations have been strongly recommended to aid postpartum weight reduction for mothers with previous GDM, subsequently reducing their risk of developing obesity and T2D. This study, informed by a previous pilot study, aims to determine the effectiveness of a pragmatic pregnancy and postpartum lifestyle modification program for overweight women with previous GDM (PAIGE2) to reduce body weight at 12 months postpartum.

**Methods/design:**

This paper summarizes the protocol for the PAIGE2 study, which has been developed based on results from a pilot study (PAIGE). A six center, two parallel arm, 12-month, randomized controlled trial will be conducted across Northern Ireland and the Republic of Ireland (3 centers each), involving 340 women with GDM and body mass index ≥25 kg/m^2^ recruited during pregnancy. The lifestyle intervention involves a one-hour virtual educational program (to take place at 32-36 weeks gestation). Postpartum, the intervention will include monthly phone calls, weekly motivational text messages, weekly step counts, and referral for three months to a Commercial Weight Management Organization (Slimming World). The control arm will receive usual care as offered by the local maternity hospital. The primary outcome is weight loss at 12 months postpartum. Study visits for anthropometric and clinical measurements, fasting blood samples, questionnaires pertaining to health, wellbeing and physical activity will take place at 6 weeks, 6- and 12-months postpartum. Focus groups will be conducted with intervention mothers’ post-intervention to determine the acceptability of the study design including utility of a Commercial Weight Management Organization, feasibility of remote patient contact, family involvement and patient satisfaction.

**Discussion:**

The PAIGE2 study will address the gaps in previously conducted research and, if positive, has the potential to have major public health implications for the prevention of future GDM and subsequent T2D.

**Clinical trial registration:**

https://clinicaltrials.gov/ct2/show/NCT04579016?term=NCT04579016&draw=2&rank=1, identifier NCT04579016.

## 1 Introduction

Gestational diabetes mellitus (GDM), classified as glucose intolerance with first recognition or onset during pregnancy, is a global health problem with reported prevalence rates of 2-18% (1, 2). A major compounding factor is excessive maternal weight and current estimates suggest that 50% of women of childbearing age are either overweight or obese (3). Women with GDM have a greater than 50% risk of developing GDM again in a future pregnancy and a 7-fold increased risk of type 2 diabetes (T2D) compared to women without GDM (4). Being overweight during pregnancy can significantly increase the risk of miscarriage, GDM, pre-eclampsia, macrosomia, instrumental delivery or caesarean section (3). Excessive gestational weight gain and postpartum weight retention are also recognized risk factors for chronic obesity (5). At 6-18 months postpartum, between 15 to 20% of women have retained a minimum of 5 kg body weight compared with pre-pregnancy body weight (6). GDM risk factors are comparable to those of T2D and the metabolic syndrome, and in the long-term, weight management may reduce T2D risk, obesity, coronary heart disease and some cancers (7).

In addition to the maternal health implications of GDM and obesity, babies born to mothers with GDM display increased rates of macrosomia, congenital malformations, admission to intensive care and fetal death (8, 9). In the longer term, these children are at greater risk of subsequent obesity and T2D (10–13). Most recently, a 10 year follow up of 4747 mother-offspring dyads from the Hyperglycemia and Adverse Pregnancy Outcome Study showed that GDM was associated with 2-3-fold higher rates of obesity in the offspring, controlled for confounding factors, with major implications for future generations (14). In a study by Catalano and colleagues, maternal pre-gravid body mass index (BMI) was the greatest predictor of childhood obesity, “independent of maternal glucose status or weight” (15).

Lifestyle modification can inhibit or impede the development of T2D in high risk non-pregnant persons (16), whereby a meta-analysis reported a relative risk of T2D development of 0.55 (0.44-0.69) with modest weight loss (17). Nutrition and physical activity collectively appear to be more successful than nutrition only in promoting weight loss post-delivery (18), and the addition of individual or group counselling in combination with written and telephone contact or diet and exercise diaries may be beneficial (19). An appraisal commissioned by the UK National Institute for Health and Care Excellence (NICE) determined that studies after childbirth are urgently required on the cost-effectiveness of weight management interventions and whether it is possible to prevent T2D (20, 21). Additionally, no consensus has been reached as to the individual constituents of the ‘optimal’ postpartum intervention, with many queries regarding the role of diet versus physical exercise, group versus individualized therapy, and the use of internet technology remaining unanswered (22–26).

In the Diabetes Prevention Program (DPP) 10-year follow up, it was reported that although women with a history of GDM experienced less weight loss compared with the general DPP population, they experienced a 35% decrease in T2D development as a result of an intensive lifestyle intervention (27). However, other papers have indicated poor diet quality and low physical activity levels amongst women with previous GDM (28–31). A recent systematic review (32) identified 10 randomized controlled trials (RCTs) of postnatal behavioral interventions in women with previous GDM. Only four RCTs found a significant result for body weight or BMI (33–36). None of those four RCTs showed a significant decrease in T2D incidence, however the pooled incidence on meta-analysis was significant (-5.02 per 100; 95% CI -9.24; -0.80). Most interventions addressed both nutrition/diet and physical activity (29, 30, 33–35, 37–39); one focused solely on physical activity with no effect on insulin resistance or body weight (40) and one exclusively addressed diet (36), finding an effect on body weight and 2-hour glucose but a non-significant reduction for fasting glucose. In summary, the authors stated that they were not able to suggest a particular intervention for T2D prevention in women with previous GDM.

There is increasing evidence that step count-based programs are successful in instigating increased ambulatory activity both among the general public and those with T2D for up to 6 months (41). On the other hand, the literature would suggest that the responsiveness of adults to simply wearing a pedometer is minimal and likely short-term (42, 43), indicating that additional support is needed to enable sustainable behavior change.

The Gestational Diabetes’ Effects on Moms cluster RCT (44) showed that a DPP-derived lifestyle intervention moderately “reduced postpartum weight retention and improved physical activity in women with a recent history of GDM” (44). These women had telephone sessions between 6-24 weeks postpartum and were urged to set weekly physical activity and nutrition goals. The primary outcomes involved reaching pre-gravid weight (if pre-gravid BMI < 25.0 kg/m^2^) or losing 5% of pre-gravid weight (if BMI ≥ 25.0 kg/m^2^). Overall, “significantly more women in the intervention group met the weight loss goal and displayed larger increases in physical activity levels compared with the usual care group” (44).

Recently published Department of Health, Health Technology Assessments Position Statements and UK NICE guidelines (20, 21), have highlighted the growing problem of pregnancy-associated weight gain, with its relevance to GDM, as a major area for urgent future public health research. This issue was further highlighted by the Hyperglycemia and Pregnancy Outcome study which estimated the prevalence of GDM in Belfast, using the new International Associations for Diabetes in Pregnancy Study Groups (IADPSG) diagnostic criteria is 15.5% (45). Although there is controversy surrounding the different methods to diagnose GDM, the IADPSG criteria have been widely accepted for more than a decade and are endorsed by the World Health Organization (46). The UK National Institute for Clinical Excellence, in their Obesity Guidance (2006) (47), highlighted the importance of providing frequent and continuing support for behavior change, to reduce weight gain, and encourage weight loss and subsequently weight maintenance. NICE also recognizes the position of Commercial Weight Management Organizations (CWMOs) which follow guidance criteria for best practice. The UK cross-departmental government report ‘Healthy Weight, Healthy Lives’ acknowledged the important role of the commercial sector and other providers in “ensuring more people can access effective services in order to increase cost-effective provision of health service capacity” (48). An RCT involving 740 subjects compared a range of commercial primary care led 12-week weight reduction programs in those with obesity across a Primary Care Trust in Birmingham, England (49). Of the data available for 658 (88%) participants at the end of the programs, and 522 (70.5%) participants at one year, the results showed that all programs aided significant weight loss from baseline to the end of the program (between 1.37-4.43kg) and all achieved significant weight loss at one year (excluding general practice and pharmacy provision).

Following a feasibility study in 2006, one CWMO (Slimming World) has been operating in collaboration with Primary Care Trusts and National Health Service (NHS) Trusts, proposing a subsidized plan to provide free membership for 12 weeks to patients referred by their health care professionals. This project, involving 34,271 patients, was recently audited, and showed an average percentage weight change of -4.0% with a mean number of sessions attended of 8.9. For patients attending at least 10-12 sessions (n=19,907 or 58.1%), the mean percentage weight change was -5.5%. Weight gain was reduced in 92.1% of referred patients. The report concluded that referral to a CWMO is a feasible alternative for NHS weight management strategies, which accomplishes clinically effective weight loss (50). Slimming World makes provision for specific groups including those with T2D, cardiovascular disease and pregnancy, and often collaborates with health care professionals. In addition, whilst weight management and dietary choices are strongly emphasized, there is a graded approach regarding advice for physical activity. An online survey including 590 members attending Slimming World up to 2 years postpartum observed that “43% of respondents had reached their pre-pregnancy weight, whilst 41% indicated they now weighed less than before they were pregnant” (51). This data, although self-reported and in a possibly unrepresentative sample of responders, suggest that the program does help postpartum women to implement healthy lifestyle behaviors, lose weight and increase their self-esteem.

After consideration of the collated evidence and recommendations from governing bodies, a pragmatic pilot multicomponent randomized postpartum lifestyle modification program for overweight women with previous GDM (PAIGE) in combination with the CWMO Slimming World and the NHS was conducted between 2013-2014 (52). The results showed a significant weight reduction at 6 months among 31 women in PAIGE compared with the 29 women receiving routine care (means 3.9 *vs* 0.7 kg respectively; p=0.02) supporting its clinical utility. A larger, definitive trial was recommended to confirm the validity of the intervention. Importantly, the intervention would seek to investigate whether a lifestyle change program for overweight and obese women with previous GDM (delivered in combination with Slimming World and the NHS), could be effective in achieving weight loss, and in the longer term, potentially a reduction in the risk of T2D.

## 2 Study objective

The objective of this study is to investigate the impact of a pragmatic lifestyle intervention during, and post pregnancy compared with routine care, on weight reduction at 12 months postnatal in overweight women with previous GDM (PAIGE2). The primary outcome will be the percentage maternal weight loss over a 12-month period. Secondary outcomes will include maternal glucose tolerance, anthropometry, number of steps per day, and several questionnaires detailing physical activity and health and wellbeing, to assess clinical, anthropometric and lifestyle- related health and well-being measures. This protocol follows the reporting guidelines of the Standard Protocol Items: Recommendations for Interventional Trials (SPIRIT) Checklist and World Health Organization Trial Registration Data Set (**Supplementary Materials 1**, **2**).

## 3 Methods and analysis

### 3.1 Design

#### 3.1.1 Pilot study

The study has been designed and developed based on results of a recently completed pilot RCT (PAIGE) (52). The study, which was informed by focus groups, clearly highlighted the demands on these women and the key issues to consider in future lifestyle interventions, including the need for simplicity, to involve partners/and or families, to consider supportive care and be open to partnership between CWMOs and NHS. The PAIGE2 study, whilst having similar overall objectives to PAIGE, has been specifically modified to reflect the information gained from PAIGE participants and in particular the challenges of the women in the postnatal period. As part of the initial PAIGE pilot, study focus groups were carried out with participants who completed the study. As a direct consequence of this feedback, PAIGE2 has been designed to make it easy for women to take part and to remain in the study, with recruitment now taking place during pregnancy at routine review clinics rather than at 6–8-week follow-up visit, which was challenging for women to attend and had high attrition rates.

The PAIGE2 research proposal has been reviewed by women with GDM during a recent pregnancy prior to submission thus ensuring that researchers complete the circle of patient involvement in the proposal. In addition, service users and the public will be closely involved in the study and women with a history of GDM will be invited to sit on the Project Board. The involvement of users within the research process will be an important guide to local researchers in finalizing wording for patient information sheets, local recruitment strategies and in relation to supporting women while on the intervention.

#### 3.1.2 Study setting

A six center, two parallel arm, randomized controlled trial, with an equal allocation ratio of intervention to control group participants will be conducted. The six centers will include antenatal-metabolic clinics at the Royal Jubilee Maternity Service, Belfast; Ulster Hospital, Dundonald; Antrim Area Hospital; Our Lady of Lourdes Hospital, Drogheda; Sligo University Hospital and Letterkenny University Hospital.

#### 3.1.3 Recruitment

All pregnant women with a BMI ≥25 kg/m^2^, recently diagnosed with GDM using IADPSG criteria (45) (either by an oral glucose tolerance test (OGTT) or a high random blood glucose result) around/between 28-30 weeks gestation, will be invited to join the study. Following a positive GDM diagnosis, woman will be referred to the joint metabolic antenatal clinic. A member of the woman’s usual multidisciplinary diabetes care team will seek the woman’s permission for her to be contacted by the PAIGE2 research team *via* a contact number provided by the woman. Women will be provided with an information sheet informing them of the study by a member of the usual diabetes care team. Women will be advised to take it home, read it thoroughly and speak to family and friends before deciding whether to participate.

Before their routine 32–36-week gestation antenatal diabetes clinic appointment, women will be contacted by a member of the PAIGE2 research team *via* the contact number provided to the diabetes care team, to answer any additional questions and confirm their interest in taking part. The participants will be informed that the study is designed to explore the feasibility of a pragmatic lifestyle intervention customized to the postnatal period, and if participants meet the study inclusion criteria (listed below), they will be randomized either to the lifestyle intervention or to usual care.

Informed consent will take place approximately 2-6 weeks after the patient is first given a patient information sheet, by a member of the PAIGE2 research team. The PAIGE2 researcher will ensure that the participants have read and understood the participant information sheet, offer further explanation, if necessary, confirm eligibility and obtain written consent prior to their enrolment in the PAIGE2 study. Only after consent has been provided by the women, will any member of the PAIGE2 research team have access to the woman’s health information. If the women have a partner, they will be given the opportunity to be involved in the study, subject to written consent.

#### 3.1.4 Randomization

To streamline the organization of the intervention, economize on staff time and avoid contamination effects, women will be randomized to treatment group according to the week they attend for review at the joint diabetes antenatal clinic at 32-26 week’s gestation. Delivery of the intervention or control program will take place weekly at each site in person or *via* telephone or video call. Weeks will be allocated at random, using a restricted randomization developed earlier. To minimize the risk of manipulation, allocations will only be made available to recruitment centers on the morning of the visit in sufficient time for necessary administrative arrangements to be made. Women who agree to participate at 32–36-week gestation but who did not subsequently turn up for their postnatal follow-ups must be considered as having been recruited but lost to follow-up. Due to the nature of the intervention, the study cannot be blinded. The number of women randomized to each schedule will be reviewed regularly by the trial statistician to ensure that approximately equal numbers are assigned to treatment group. Should a serious imbalance develop, an additional intervention or control week will be added as appropriate.

#### 3.1.5 Inclusion criteria

Inclusion and exclusion criteria for this study are similar to that of the original PAIGE study (52).

1. Women aged ≥18 years.
2. Booking BMI ≥25 kg/m^2^ at <14 weeks gestation.
3. GDM diagnosed in the current pregnancy. GDM will be defined by the WHO (2013) criteria (46) (fasting plasma glucose (FPG) ≥ 5.1 mmol/l or 1h postprandial glucose ≥ 10.0 mmol/l or 2h plasma glucose ≥8.5 mmol/l). Women with a history of GDM directly prior to the current pregnancy, and who were given lifestyle advice and were performing self-monitoring of blood glucose from early pregnancy, will also be included if capillary glucose monitoring exceeds target values (fasting ≥ 5.3 mmol/l (95 mg/dl) or 1h postprandial plasma glucose ≥ 7.8 mmol/l (140 mg/dl)) thus negating the need for an OGTT (52).
4. Women will only continue to be included in the study if their fasting plasma glucose the morning after delivery or at 6 weeks postpartum is either normal or consistent with impaired fasting plasma glucose outside of pregnancy (i.e., <7 mmol/l). (If the woman requests an earlier discharge that does not allow time for the immediate post-delivery FPG test, she will be offered an appointment to return to the maternity unit within 4-6 weeks of birth for retesting) (52).

#### 3.1.6 Exclusion criteria

1. Pregnancy in which an anomaly has been detected on the 20-week fetal anomaly scan.
2. History of diabetes outside of pregnancy or FPG ≥7 mmol/l the morning after delivery or at 6 weeks postpartum (these women will be excluded and referred to the local diabetes care team)
3. History of heart, liver, or chronic renal disease.
4. Medications that adversely affect glucose tolerance (e.g., steroids).
5. Inability to participate in moderate physical activity outside of pregnancy.
6. Moderate/severe depressive illness or excess alcohol consumption.
7. Inability to understand adequately verbal explanations or written information in English, or special communication needs.
8. Women who are planning another pregnancy within the next 12 months following delivery.
9. Women who are intending to attend a CWMO program after pregnancy.
10. Exclusion criteria for early postpartum glucose testing will include massive postpartum hemorrhage, steroid treatment prior to delivery or patient refusal.

#### 3.1.7 Sample size

Assuming the same variability in weight loss as was observed in the pilot study (52) (standard deviations of 7.0 and 3.9 kg in the intervention and control groups, respectively) and a 40% drop out rate (including women diagnosed with overt diabetes on the basis of a fasting glucose sample postnatal), it is estimated that a trial of 340 women should yield 200 evaluable subjects (100 in each of the intervention and control groups) which will be sufficient to detect a 3kg difference in maternal weight at 12 months after delivery with >90% power at the 5% level of significance.

### 3.2 Interventional methods

#### 3.2.1 Intervention group

The education program will be delivered in person or by telephone or video call. The program will comprise one session lasting 60 minutes, delivered either in the hospital setting or by telephone or video call by a research nurse or dietitian both trained in motivational interviewing. The content will be underpinned by the principles used in the DPP (53) and will be designed to augment the further information received from the CWMO. The educational program will take account of socioeconomic status and ethnicity and will be specifically customized to post-pregnancy as informed by previous focus groups and guided by the existing literature, contextual factors impacting on nutrition and physical activity.

The intervention program will broadly be divided into the same information as the original PAIGE study (52): one third of the time talking about the “causes, consequences, difficulties, timelines and implications of GDM and overweight/obesity” (52), one third discussing advice regarding a healthy nutritional approach (52), and the remaining third tackling the supposed success of physical activity as an alternative treatment for the prevention of T2D and potential future GDM, including recommendations, barriers and strategies (52). The program will be modelled on the person-centered philosophy and techniques created to stimulate lifestyle modification and self-management in keeping with the Medical Research Council’s framework for complex interventions (54), updated in 2021 (55).

At 6-8 weeks, all postpartum mothers in the intervention group will be offered a free 12-week voucher for a CWMO (Slimming World). This voucher can be used by the participant to attend a local or online group of their choosing, run by Slimming World, for 12 consecutive weeks. Slimming World is group-based, and the mothers can enroll at any time. Meetings convene in community locations or online and are usually 90 minutes in duration. Mothers can also avail of a website, magazines and one to one telephone support from a Slimming World consultant or other Slimming World members. Like the original PAIGE study (52), the women in the study will be urged to consume foods predominantly with low energy density to reach satiety, including foods high in nutrients such as calcium and fiber, with restricted quantities of high energy dense foods. Principal behavior strategies used in Slimming World include weekly weighing, group support and commendation for weight loss, and sustained dedication regardless of amount of weight lost. Individualized support, if required, involves self-monitoring of diet and emotional eating, for and against assessments, visualization techniques and individual diet plans. Individual weight loss goals will be set by each mother. Physical activity is also promoted, with gradual increase to 30 minutes of moderate-to-intense activity 5 days per week. Slimming World’s theoretical underpinning is modelled on motivational interviewing and transactional analysis.

#### 3.2.2 Control group

Women in the control arm will receive routine antenatal care as offered by their individual maternity hospital. When the study is concluded, the control group will be offered referral to Slimming World (12 consecutive weeks) free of charge and will be given access to the educational resources from the one-hour educational session.

#### 3.2.3 Study timepoints

**Figure 1:**
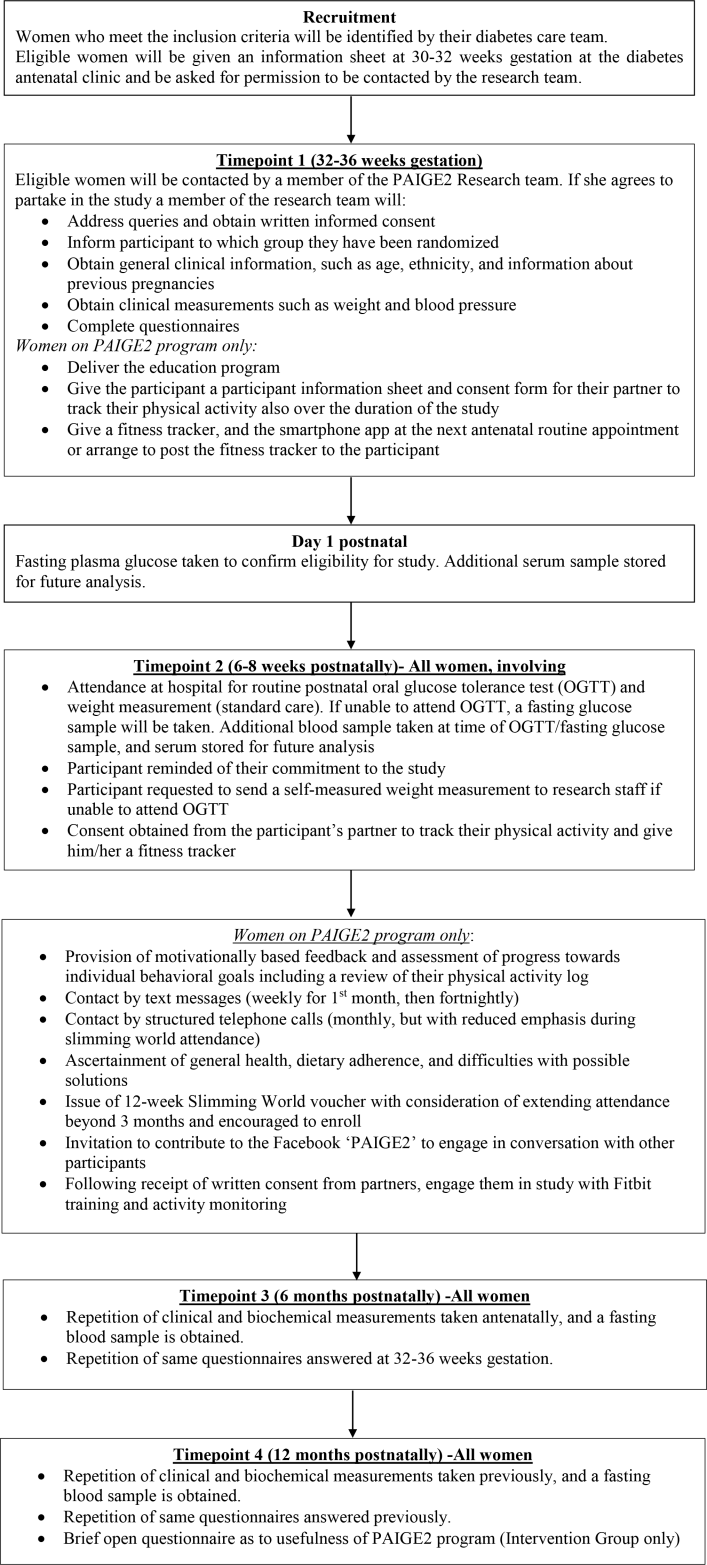
Study Flowchart.

The study will involve four data collection appointments with the participants, as depicted in **Figure 1**.

##### 3.2.3.1 Timepoint one

Timepoint one will be the first study contact and will take place over the phone before 32-36 weeks gestation. Informed consent will be obtained at this point, followed by collection of baseline clinical and demographic data and questionnaire completion. Relevant clinical and obstetric data will be abstracted from patient notes and recorded in study specific case report forms.

Those women randomized to the lifestyle intervention will be enrolled into the study in small groups where they will receive an educational program lasting around one hour (as described above). They will also be given an activity tracking device (Fitbit) to allow them to become familiar with this technology during pregnancy. This device will either be posted to participating women or collected from research staff at the women’s next antenatal appointment. The education program will be led by a PAIGE2 research nurse or dietitian trained in motivational interviewing and will cover topics including GDM as a risk factor for future disease, healthy eating, physical activity, fitness tracker use, methods to reduce the risk of diabetes in subsequent pregnancies in the longer term, and ideas for maximizing peer support. The program will be loosely based on that used in the DPP (53) and designed to augment rather than duplicate the information the women will receive from Slimming World.

##### 3.2.3.2 Timepoint two

Timepoint two is the second study contact and will take place at around 6-8 weeks postnatal when women attend routinely for OGTT. Women will be contacted at this visit to confirm their continuing willingness and eligibility to take part in PAIGE2. They will be weighed (or if this visit is not possible, they will be contacted and asked to weigh themselves and measure their waist circumference at home and forward their self-measured weight to the research staff). Alternatively, if they do not provide an OGTT, a fasting plasma glucose sample will be taken. All mothers (whether they provided an OGTT or fasting plasma glucose sample) will also provide a fasting blood sample to measure lipids and renal function. An additional serum sample will be stored for future analysis. This weight will function as a baseline measurement. The women will also be invited to upload their capillary glucose monitoring (glucometer) data during pregnancy to a secure website for examination in relation to other pregnancy-related variables such as physical activity. Those in the intervention will be offered a 12-week Slimming World voucher (free to the individual) to be completed within the 12-month period. This voucher can be used by the person to attend a group of their choosing, for 12 consecutive weeks. Women in the treatment group will be contacted by text messages (weekly for first month and then fortnightly) to provide motivation based feedback and to assess progress towards behavioral goals including a review of the step count, sleep activity and food intake.

Subsequent contact with the women will be by text or telephone a call. The frequency of contact by text message will be weekly for the initial month (to persuade the women to sign up for the CWMO) and then fortnightly for the remainder on the study. Structured telephone calls will occur monthly, and include information of physical activity, barriers to achieving their diet and physical activity goals, as well as suggested solutions. Participants who are successful during the first three-month period of enrolment with Slimming World (gauged by attending at least 9 out of 12 sessions or demonstrated weight loss), will be offered a second funded three-month membership to maximize any benefits obtained during the program. After the second free three-month Slimming World program, women will be able to continue to attend Slimming World for the rest of the study if they desire, at their own discretion. Partners of the women in the intervention arm will also be invited to participate in the trial. Partners consent will be obtained either by post or in person at the same 6-8 post gestational timepoint, and they will subsequently be offered a fitness tracking device like that of their partner. Intermittent contact *via* text will be made with the partner to feedback on their physical activity.

##### 3.2.3.3 Timepoints three and four

At timepoints 3 and 4, the third and final study contacts, all women will be weighed (or asked to weigh themselves at home and forward their self-recorded weight to the research staff). They will also have an additional (fasting) blood sample taken, along with a measurement of their waist circumference and their blood pressure. In addition, they will be asked to complete the same questionnaires they answered at 32-36 weeks gestation.

#### 3.2.4 Measurable outcomes

A summary of the several measurable outcomes of this study can be found in **Table 1**.

**Table 1:** Measurable outcomes and their collection timepoints (TP).

Measurable outcome	Contact point measurement			
	*TP1*	*TP2*	*TP3*	*TP4*
Clinical history	x			
Anthropometric measurements (body weight, waist circumference, BMI)	x	x	x	x
Biochemistry measurements*		x	x	x
Step count (using Fitbit wearable fitness tracker) **	x	x	x	x
*Questionnaires*				
International Physical Activity (IPAC) 7day questionnaire	x	x	x	x
Risk Perception Survey for Developing Diabetes (RPS-DD)	x	x	x	x
Health and Wellbeing SF-12 Version 2	x	x	x	x
Edinburgh Postnatal Depression Scale		x	x	x
Motivation to Change Questionnaire	x	x	x	x
Exercise Self-Efficacy Questionnaire	x	x	x	x

TP1, Timepoint1 (32-36 weeks gestation); TP2, Timepoint 2 (6 weeks postpartum); TP3, Timepoint 3 (6 months postpartum); TP4, Timepoint 4, (12 months postpartum).

*Additionally taken initially day 1 postnatal, including fasting glucose, total cholesterol, triglycerides, high density lipoproteins, low density lipoproteins, non-high-density lipoproteins, cholesterol/high density lipoprotein ratio, urea and electrolytes.

** Used daily throughout the 12 months, also includes partners step counts (if applicable).

##### 3.2.4.1 Primary endpoints

1. Weight at 12 months (accounting for any difference in weight at baseline between the intervention and control groups)

- Body weight: Women will be weighed (to the nearest 0.1kg) when they attend their 6-8 weeks postnatal appointment performed as part of routine standard care and will act as the baseline measurement. If they are unable to attend the department for this, the women will be asked to weigh themselves at home 6-8 weeks postnatal and send their self-measured weight in a text message to the research staff.
- Height (to the nearest 0.1cm), to calculate BMI (kg/m^2^).

##### 3.2.4.2 Secondary outcomes

1) Fasting glucose

- Day 1 postnatal (Inpatient in hospital following delivery): A fasting venous blood sample will be taken for glucose measurement and a serum sample will be stored for future analysis.
- Timepoint 2 (6-8 weeks postnatal): Participants will confirm eligibility by an OGTT when they attend their postnatal routine standard care appointment, or alternatively a fasting plasma glucose sample will be taken, along with a fasting blood sample to measure lipids and renal function. An additional serum sample will be stored for future analysis.
- Timepoint 3 (6 months postnatal) and timepoint 4 (12 months postnatal): A maternal blood sample will be taken for fasting plasma glucose, lipids, and renal function. An additional serum sample will be stored for future analysis.

2) Waist circumference
Waist circumference (midpoint between lower costal margin and iliac crest) (cm), hip circumference (cm).
3) Step counts, change from baseline to 12 months (intervention group only).
The Fitbit tracker will allow women and their partners to upload their daily data to a secure dedicated spreadsheet. Women will choose what form of activity to engage in such as walking, dancing, aerobic activity etc., and will be encouraged to set individualized step count goals based on their baseline step count. Accumulation of all activity across the day will be promoted. Sedentary participants will be encouraged to increase their activity levels by a minimum of 3,000 steps per day, equivalent to approximately half an hour of walking (56). Goal attainment will be encouraged using numerical objectives, for example, increasing steps by 500 steps per day every fortnight and encouraged to repeat this method for each new goal (52). They will also be encouraged to wear their fitness tracker daily to view and track their activity and will be prompted to record this by uploading the data onto a secure website.
4) Questionnaires

- Physical activity will also be recorded at all timepoints using two questionnaires. The International Physical Activity Questionnaire (IPAQ) (57) will provide a record of both light and moderate-to-vigorous intensity activities carried out for 10 uninterrupted minutes. The IPAQ has provided sufficient validity in comparison with accelerometer data in the UK. An exercise self-efficacy questionnaire will provide a measure of participants’ belief in their ability to exercise and will identify barriers including motivation and willingness to exercise in certain situations (58).
- The Risk Perception Survey for Developing Diabetes (RPSDD) (59), adapted for women with a history of GDM, is a self-reported or telephone interview questionnaire used to evaluate risk perception and modifiers of risk perception among women with a history of GDM who have not developed T2D postpartum (59). The instrument comprises 4 scales assessing “modifiers of risk perception, including patients’ knowledge of diabetes risk factors, perceptions of personal control, optimism about developing diabetes and other diseases, and perceptions of the benefits and barriers of preventive behaviors” (59). The survey takes approximately 5 minutes, also includes two scales evaluating actual risk perception.
- Psychological measures and health and wellbeing will be measured at all timepoints using the 12-Item Short-Form (SF-12v2) Health Survey (60).
- Postpartum depression will be assessed using the Edinburgh Postnatal Depression Scale at timepoints 2-4 (61).
- Motivation for change will be measured at all timepoints by asking two questions devised by the team. For women in the intervention group motivation for change will be measured both before the delivery of the one-hour educational session and after, to assess the influence of the session on motivation to change.

All the questionnaires have established reliability and validity.

##### 3.2.4.3 Other outcomes

1. Compliance: Slimming World records include attendance, quantity of sessions and weight at first and last sessions (intervention group only).
2. Blood Pressure: Blood pressure (automated, average of last 2 of 3 measurements taken in non-dominant arm after being seated for 5 minutes).
3. Follow up analysis of those women who have a further pregnancy within 12 months to document: BMI at booking, development of GDM and birth weight.
4. Partner weekly step count (if applicable)

#### 3.2.5 Data collection and management

All instruments will be administered to participants by the researchers and all data, including that obtained from patient notes to record usual care/treatment, will be collated on a study-specific case report form starting at the time of recruitment. Data will be maintained and managed by the PAIGE2 research team within the Belfast Health and Social Care Trust (sponsor). OGTTs and fasting blood samples will be analyzed and stored within the Regional Centre for Endocrinology and Diabetes, Royal Victoria Hospital, Belfast. Trial recruitment will be monitored by the Principal Investigator and a project manager will coordinate all aspects of the trial and manage and analyze all data under their supervision. An educational clinical psychologist will provide advice on the psychological aspects of the study including motivational interviewing and a statistician will oversee randomization and data analysis. All paper and electronic records relating to the study will be retained for 5 years.

### 3.3 Data analysis

#### 3.3.1 Statistical analysis

Primary and secondary outcomes at 12 months will be compared between the intervention and control groups using analysis of covariance to adjust for any differences at baseline and so produce an estimate of the intervention effect with 95% confidence limits. Adjustment will also be made for recruitment center and any characteristics relevant to the outcome which, by chance, show imbalance between groups. Prior to analysis, logarithmic transformations will be applied to those secondary outcome variables whose distributions show marked positive skew. A correction will be applied to standard errors of intervention effect estimates to make allowance for the clustered nature (by week) of the randomization, although analysis of the pilot study (which used the same randomization procedure) indicated that this will make little difference to the findings. To help assess the impact of non-response, sensitivity analyses will be conducted on key outcomes by multiple imputation of missing values by chained estimating equations using the commands provided in the Stata package (StataCorp, College Station, TX). We will use this approach to impute 10 complete datasets and analyze them appropriately to provide results which take account of missingness. For key outcome variables, this analysis will be reported in addition to the complete case analysis. Analysis will be by intention to treat.

#### 3.3.2 Focus groups

Women from the intervention group during their final visit will be invited to take part in focus groups (arranged for up to ten participants per group) to feedback on their experiences of the program and to suggest how the program might be improved. This feedback about will be their experience within the trial, including the research tools used, Slimming World referral, fitness tracker use, and the educational session will be important to inform any future trial design or future roll out of the intervention. Focus group sessions will be recorded using voice recorders with transcripts anonymized prior to analysis of feedback. Focus group participation will be optional and those women who take part will receive a multistore gift voucher to reimburse them for their time. All women in the intervention group will be asked to complete a questionnaire regarding feedback for the study.

## 4 Discussion

The PAIGE2 study is a large-scale investigation into the implementation of an evidence-based pragmatic pregnancy and postpartum lifestyle intervention for overweight women with GDM. The study has been informed from the results of a pilot trial (PAIGE) (52), which provided promising results in postnatal weight reduction for mothers over a 6 month period. The study was developed taking account of the views of mothers with GDM, alongside a Project Steering Committee and patient group. The primary outcome of the PAIGE2 study is weight loss at 12 months postpartum.

A potential limitation of this study may be the differing nature of the healthcare systems in Northern Ireland and the Republic of Ireland. Obtaining ethical approval from the three Republic of Ireland hospital sites may prove difficult as the Republic of Ireland has no central ethics office/structure. However, the cross-border collaboration demonstrated in this trial should provide an evidence-based evaluation on the potential impact of a pregnancy and post-pregnancy lifestyle intervention for overweight mothers with GDM in an all-Ireland context. The cross-border approach should also identify optimum service delivery for successful implementation and impact of a lifestyle intervention for weight loss and GDM on the island of Ireland. Additional limitations may include participation-related factors, such as enrolment in Slimming World etc., which may influence their progress within the intervention. In addition, there may be some disparity between self-reported *vs* clinically measured body weight across the timepoints, and use of the fasting glucose immediately post-partum compared with 6 weeks postnatally to exclude diabetes. However, these will be minimized as much as possible by the research team.

The results from this study will be distributed to healthcare professionals through conference presentations and peer-reviewed journals to add to the current literature and evidence base for effective interventions to address this major public health issue.

## Ethics statement

The studies involving human participants were reviewed and approved by Research Governance and approval was obtained from the Belfast Health and Social Care Trust, South Eastern Health and Social Care Trust and Northern Health and Social Care Trust (18/NI/0228). The patients/participants provided their written informed consent to participate in this study.

## Author contributions

DM, UG, HW, CP and LC contributed to the development of study protocol. BJK wrote the protocol manuscript. All authors contributed to the article and approved the submitted version.

## References

[1] Coustan DR Lowe LP Metzger BE Dyer AR . The hyperglycemia and adverse pregnancy outcome (HAPO) study: Paving the way for new diagnostic criteria for gestational diabetes mellitus. Am J Obstet Gynecol (2010) 202(6):654.e1–6. doi: 10.1016/j.ajog.2010.04.006 PMC2897007 20510967

[2] Ferrara A . Increasing prevalence of gestational diabetes mellitus: A public health perspective. Diabetes Care (2007) 30 Suppl 2:S141–6. doi: 10.2337/dc07-s206 17596462

[3] Scott-Pillai R Spence D Cardwell CR Hunter A Holmes VA . The impact of body mass index on maternal and neonatal outcomes: A retrospective study in a UK obstetric population, 2004-2011. BJOG (2013) 120(8):932–9. doi: 10.1111/1471-0528.12193 23530609

[4] Bellamy L Casas JP Hingorani AD Williams D . Type 2 diabetes mellitus after gestational diabetes: A systematic review and meta-analysis. Lancet (2009) 373(9677):1773–9. doi: 10.1016/S0140-6736(09)60731-5 19465232

[5] Gore SA Brown DM West DS . The role of postpartum weight retention in obesity among women: A review of the evidence. Ann Behav Med (2003) 26(2):149–59. doi: 10.1207/S15324796ABM2602_07 14534032

[6] Calfas KJ Marcus BH . Postpartum weight retention: A mother’s weight to bear? Am J Prev Med (2007) 32(4):356–7. doi: 10.1016/j.amepre.2007.01.008 17383569

[7] Mestman G . Long term implications for hyperglycaemia in pregnancy. In: McCance DR Maresh M Sacks DA , editors. A practical manual of diabetes in pregnancy. Hoboken, NJ: Wiley-Blackwell (2010). p. 242–50.

[8] Ramachenderan J Bradford J McLean M . Maternal obesity and pregnancy complications: A review. Aust N Z J Obstet Gynaecol (2008) 48(3):228–35. doi: 10.1111/j.1479-828X.2008.00860.x 18532950

[9] Lewis G . Confidential enquiry into maternal and child health. saving mothers’ lives: Reviewing maternal deaths to make motherhood safer 2003-2005. Sheffield, UK: London: CEMACH (2007).

[10] Boney CM Verma A Tucker R Vohr BR . Metabolic syndrome in childhood: association with birth weight, maternal obesity, and gestational diabetes mellitus. Pediatrics (2005) 115(3):e290–6. doi: 10.1542/peds.2004-1808 15741354

[11] Dabelea D Hanson RL Lindsay RS Pettitt DJ Imperatore G Gabir MM . Intrauterine exposure to diabetes conveys risks for type 2 diabetes and obesity: A study of discordant sibships. Diabetes (2000) 49(12):2208–11. doi: 10.2337/diabetes.49.12.2208 11118027

[12] Mingrone G Manco M Mora ME Guidone C Iaconelli A Gniuli D . Influence of maternal obesity on insulin sensitivity and secretion in offspring. Diabetes Care (2008) 31(9):1872–6. doi: 10.2337/dc08-0432 PMC2518362 18535193

[13] Whitaker RC . Predicting preschooler obesity at birth: The role of maternal obesity in early pregnancy. Pediatrics (2004) 114(1):e29–36. doi: 10.1542/peds.114.1.e29 15231970

[14] Perak AM Lancki N Kuang A Labarthe DR Allen NB Shah SH . Associations of maternal cardiovascular health in pregnancy with offspring cardiovascular health in early adolescence. JAMA (2021) 325(7):658–68. doi: 10.1001/jama.2021.0247 PMC7887661 33591345

[15] Catalano PM Farrell K Thomas A Huston-Presley L Mencin P de Mouzon SH . Perinatal risk factors for childhood obesity and metabolic dysregulation. Am J Clin Nutr (2009) 90(5):1303–13. doi: 10.3945/ajcn.2008.27416 PMC2762159 19759171

[16] Gillies CL Abrams KR Lambert PC Cooper NJ Sutton AJ Hsu RT . Pharmacological and lifestyle interventions to prevent or delay type 2 diabetes in people with impaired glucose tolerance: Systematic review and meta-analysis. BMJ (2007) 334(7588):299. doi: 10.1136/bmj.39063.689375.55 17237299 PMC1796695

[17] Yamaoka K Tango T . Efficacy of lifestyle education to prevent type 2 diabetes: A meta-analysis of randomized controlled trials. Diabetes Care (2005) 28(11):2780–6. doi: 10.2337/diacare.28.11.2780 16249558

[18] Berger AA Peragallo-Urrutia R Nicholson WK . Systematic review of the effect of individual and combined nutrition and exercise interventions on weight, adiposity and metabolic outcomes after delivery: Evidence for developing behavioral guidelines for post-partum weight control. BMC Pregnancy Childbirth (2014) 14:319. doi: 10.1186/1471-2393-14-319 25208549 PMC4176850

[19] Messina J Johnson M Campbell F Everson Hock E Guillaume L Duenas A . Systematic review of weight management interventions after childbirth. Sheffield, UK: ScHARR Public Health Collaboration Centre (2009).

[20] Duenas A Rawdin A Chilcott J Messina J Johnson M Campbell F . The cost-effectiveness of weight management interventions following childbirth. Sheffield, UK: ScHARR Public Health Collaboration Centre (2011).

[21] NICE . Diabetes in pregnancy: Management from preconception to the postnatal period. London, UK: National Institute for Health and Clinical Excellence (UK) (2015), Report No. NG3. 32212588

[22] Bertz F Brekke HK Ellegård L Rasmussen KM Wennergren M Winkvist A . Diet and exercise weight-loss trial in lactating overweight and obese women. Am J Clin Nutr (2012) 96(4):698–705. doi: 10.3945/ajcn.112.040196 22952179

[23] Colleran HL Lovelady CA . Use of MyPyramid menu planner for moms in a weight-loss intervention during lactation. J Acad Nutr Diet (2012) 112(4):553–8. doi: 10.1016/j.jand.2011.12.004 22709705

[24] Craigie AM Macleod M Barton KL Treweek S Anderson AS . Supporting postpartum weight loss in women living in deprived communities: Design implications for a randomised control trial. Eur J Clin Nutr (2011) 65(8):952–8. doi: 10.1038/ejcn.2011.56 PMC3154650 21559034

[25] Østbye T Krause KM Lovelady CA Morey MC Bastian LA Peterson BL . Active mothers postpartum: A randomized controlled weight-loss intervention trial. Am J Prev Med (2009) 37(3):173–80. doi: 10.1016/j.amepre.2009.05.016 PMC2774935 19595557

[26] Spahn JM Reeves RS Keim KS Laquatra I Kellogg M Jortberg B . State of the evidence regarding behavior change theories and strategies in nutrition counseling to facilitate health and food behavior change. J Am Diet Assoc (2010) 110(6):879–91. doi: 10.1016/j.jada.2010.03.021 20497777

[27] Aroda VR Christophi CA Edelstein SL Zhang P Herman WH Barrett-Connor E . The effect of lifestyle intervention and metformin on preventing or delaying diabetes among women with and without gestational diabetes: The diabetes prevention program outcomes study 10-year follow-up. J Clin Endocrinol Metab (2015) 100(4):1646–53. doi: 10.1210/jc.2014-3761 PMC4399293 25706240

[28] Jones EJ Roche CC Appel SJ . A review of the health beliefs and lifestyle behaviors of women with previous gestational diabetes. J Obstet Gynecol Neonatal Nurs (2009) 38(5):516–26. doi: 10.1111/j.1552-6909.2009.01051.x 19883473

[29] Morrison MK Koh D Lowe JM Miller YD Marshall AL Colyvas K . Postpartum diet quality in Australian women following a gestational diabetes pregnancy. Eur J Clin Nutr (2012) 66(10):1160–5. doi: 10.1038/ejcn.2012.84 22781022

[30] Persson M Winkvist A Mogren I . Lifestyle and health status in a sample of Swedish women four years after pregnancy: A comparison of women with a history of normal pregnancy and women with a history of gestational diabetes mellitus. BMC Pregnancy Childbirth (2015) 15(1):57. doi: 10.1186/s12884-015-0487-2 25884665 PMC4372034

[31] Yun S Kabeer NH Zhu BP Brownson RC . Modifiable risk factors for developing diabetes among women with previous gestational diabetes. Prev Chronic Dis (2007) 4(1):A07. 17173715 PMC1832125

[32] Pedersen ALW Terkildsen Maindal H Juul L . How to prevent type 2 diabetes in women with previous gestational diabetes? a systematic review of behavioural interventions. Prim Care Diabetes (2017) 11(5):403–13. doi: 10.1016/j.pcd.2017.05.002 28601549

[33] Nicklas JM Zera CA England LJ Rosner BA Horton E Levkoff SE . A web-based lifestyle intervention for women with recent gestational diabetes mellitus: A randomized controlled trial. Obstet Gynecol (2014) 124(3):563–70. doi: 10.1097/AOG.0000000000000420 PMC4401073 25162257

[34] Peacock AS Bogossian FE Wilkinson SA Gibbons KS Kim C McIntyre HD . A randomised controlled trial to delay or prevent type 2 diabetes after gestational diabetes: Walking for exercise and nutrition to prevent diabetes for you. Int J Endocrinol (2015) 2015:423717. doi: 10.1155/2015/423717 26089886 PMC4452189

[35] Pérez-Ferre N Del Valle L Torrejón MJ Barca I Calvo MI Matía P . Diabetes mellitus and abnormal glucose tolerance development after gestational diabetes: A three-year, prospective, randomized, clinical-based, Mediterranean lifestyle interventional study with parallel groups. Clin Nutr (2015) 34(4):579–85. doi: 10.1016/j.clnu.2014.09.005 25262459

[36] Shyam S Arshad F Abdul Ghani R Wahab NA Safii NS Nisak MY . Low glycaemic index diets improve glucose tolerance and body weight in women with previous history of gestational diabetes: A six months randomized trial. Nutr J (2013) 12:68. doi: 10.1186/1475-2891-12-68 23705645 PMC3671161

[37] Reinhardt JA van der Ploeg HP Grzegrzulka R Timperley JG . Lmplementing lifestyle change through phone-based motivational interviewing in rural-based women with previous gestational diabetes mellitus. Health Promot J Aust (2012) 23(1):5–9. doi: 10.1071/HE12005 22730940

[38] Shek NW Ngai CS Lee CP Chan JY Lao TT . Lifestyle modifications in the development of diabetes mellitus and metabolic syndrome in Chinese women who had gestational diabetes mellitus: A randomized interventional trial. Arch Gynecol Obstet (2014) 289(2):319–27. doi: 10.1007/s00404-013-2971-0 23897066

[39] Wein P Beischer N Harris C Permezel M . A trial of simple versus intensified dietary modification for prevention of progression to diabetes mellitus in women with impaired glucose tolerance. Aust N Z J Obstet Gynaecol (1999) 39(2):162–6. doi: 10.1111/j.1479-828X.1999.tb03363.x 10755770

[40] McIntyre HD Peacock A Miller YD Koh D Marshall AL . Pilot study of an individualised early postpartum intervention to increase physical activity in women with previous gestational diabetes. Int J Endocrinol (2012) 2012:892019. doi: 10.1155/2012/892019 22548057 PMC3324899

[41] Bravata DM Smith-Spangler C Sundaram V Gienger AL Lin N Lewis R . Using pedometers to increase physical activity and improve health: A systematic review. JAMA (2007) 298(19):2296–304. doi: 10.1001/jama.298.19.2296 18029834

[42] Eastep E Beveridge S Eisenman P Ransdell L Shultz B . Does augmented feedback from pedometers increase adults’ walking behavior? Percept. Mot Skills (2004) 99(2):392–402. doi: 10.2466/pms.99.2.392-402 15560326

[43] Matevey C Rogers LQ Dawson E Tudor-Locke C . Lack of reactivity during pedometer self-monitoring in adults. Meas Phys Educ Exerc Sci (2006) 10(1):1–11. doi: 10.1207/s15327841mpee1001_1

[44] Ferrara A Hedderson MM Brown SD Albright CL Ehrlich SF Tsai AL . The comparative effectiveness of diabetes prevention strategies to reduce postpartum weight retention in women with gestational diabetes mellitus: The gestational diabetes’ effects on moms (GEM) cluster randomized controlled trial. Diabetes Care (2016) 39(1):65–74. doi: 10.2337/dc15-1254 26657945 PMC4686847

[45] Pettitt DJ McKenna S McLaughlin C Patterson CC Hadden DR McCance DR . Maternal glucose at 28 weeks of gestation is not associated with obesity in 2-year-old offspring: The Belfast hyperglycemia and adverse pregnancy outcome (HAPO) family study. Diabetes Care (2010) 33(6):1219–23. doi: 10.2337/dc09-2384 PMC2875426 20215449

[46] World Health O . Diagnostic criteria and classification of hyperglycaemia first detected in pregnancy. Geneva: World Health Organization (2013). Contract No.: WHO/NMH/MND/13.2. 24199271

[47] Centre for Public Health Excellence . Obesity: The prevention, identification, assessment and management of overweight and obesity in adults and children. London: National Institute for Health and Clinical Excellence (UK (2006). 22497033

[48] Department of Health . Healthy weight, health lives: A cross-government strategy for England. London: National Institute for Health and Clinical Excellence (UK) (2008).

[49] Jolly K Lewis A Beach J Denley J Adab P Deeks JJ . Comparison of range of commercial or primary care led weight reduction programmes with minimal intervention control for weight loss in obesity: Lighten up randomised controlled trial. BMJ (2011) 343:d6500. doi: 10.1136/bmj.d6500 22053315 PMC3208022

[50] Stubbs RJ Pallister C Whybrow S Avery A Lavin J . Weight outcomes audit for 34,271 adults referred to a primary care/commercial weight management partnership scheme. Obes Facts (2011) 4(2):113–20. doi: 10.1159/000327249 PMC6444690 21577018

[51] Avery A Allan J Lavin J Pallister C . Supporting postnatal women to lose weight. Lancet (2010) 368(9542):1164–70.

[52] Holmes VA Draffin CR Patterson CC Francis L Irwin J McConnell M . Postnatal lifestyle intervention for overweight women with previous gestational diabetes: A randomized controlled trial. J Clin Endocrinol Metab (2018) 103(7):2478–87. doi: 10.1210/jc.2017-02654 29762737

[53] Knowler WC Barrett-Connor E Fowler SE Hamman RF Lachin JM Walker EA . Reduction in the incidence of type 2 diabetes with lifestyle intervention or metformin. N Engl J Med (2002) 346(6):393–403. 11832527 10.1056/NEJMoa012512 PMC1370926

[54] Craig P Dieppe P Macintyre S Michie S Nazareth I Petticrew M . Developing and evaluating complex interventions: The new medical research council guidance. BMJ (2008) 337:a1655. doi: 10.1136/bmj.a1655 18824488 PMC2769032

[55] Skivington K Matthews L Simpson SA Craig P Baird J Blazeby JM . A new framework for developing and evaluating complex interventions: Update of medical research council guidance. BMJ (2021) 374:n2061. doi: 10.1136/bmj.n2061 34593508 PMC8482308

[56] Tudor-Locke C Bassett DR Jr. How many steps/day are enough? preliminary pedometer indices for public health. Sports Med (2004) 34(1):1–8. doi: 10.2165/00007256-200434010-00001 14715035

[57] Craig CL Marshall AL Sjöström M Bauman AE Booth ML Ainsworth BE . International physical activity questionnaire: 12-country reliability and validity. Med Sci Sports Exerc (2003) 35(8):1381–95. doi: 10.1249/01.MSS.0000078924.61453.FB 12900694

[58] Sallis JF Grossman RM Pinski RB Patterson TL Nader PR . The development of scales to measure social support for diet and exercise behaviors. Prev Med (1987) 16(6):825–36. doi: 10.1016/0091-7435(87)90022-3 3432232

[59] Kim C McEwen LN Piette JD Goewey J Ferrara A Walker EA . Risk perception for diabetes among women with histories of gestational diabetes mellitus. Diabetes Care (2007) 30(9):2281–6. doi: 10.2337/dc07-0618 17575087

[60] Ware J Jr. Kosinski M Keller SD . A 12-item short-form health survey: Construction of scales and preliminary tests of reliability and validity. Med Care (1996) 34(3):220–33. doi: 10.1097/00005650-199603000-00003 8628042

[61] Cox JL Holden JM Sagovsky R . Detection of postnatal depression: Development of the 10-item Edinburgh postnatal depression scale. Br J Psychiatry (1987) 150:782–6. doi: 10.1192/bjp.150.6.782 3651732

