# Supplementary material for: A pragmatic lifestyle intervention for overweight and obese women with gestational diabetes mellitus (PAIGE2): A parallel arm, multicenter randomized controlled trial study protocol
Source: Front Clin Diabetes Healthc. 2023 Mar 24;4:1118509. doi: 10.3389/fcdhc.2023.1118509 (PMC10080069; doi:10.3389/fcdhc.2023.1118509)
Supplement: Supplementary file 1 [file Table_1.docx]

Supplementary Material 1

**Items from the World Health Organization Trial Registration Data Set**

| **Data category** | **Information** |
| --- | --- |
| **Primary registry and trial identifying number** | ClinicalTrials.gov Identifier: NCT04579016 |
| **Date of registration in primary registry** | 1st October 2020 |
| **Secondary identifying numbers** | 18/NI/0228 |
| **Source(s) of monetary or material support** | **This project is supported by The Cross-border Healthcare Intervention Trials In Ireland Network (CHITIN), a cross-border partnership between the Northern Ireland Public Health Agency and the Republic of Ireland Health Research Board.**  **The finances for the project will be primarily managed by the Finance Department of the Belfast Health and Social Care Trust** |
| **Primary sponsor** | **Belfast Health and Social Care Trust** |
| **Secondary sponsor(s)** | N/A |
| **Contact for public queries** | **Contact name**: Professor David McCance **Address**: Royal Victoria Hospital, Belfast, United Kingdom, BT12 6BA **Telephone**: +44 (0) 7958036242 **Email**: [david.mccance@belfasttrust.hscni.net](mailto:david.mccance@belfasttrust.hscni.net) |
| **Contact for scientific queries** | **Contact name**: Professor David McCance **Address**: Royal Victoria Hospital, Belfast, United Kingdom, BT12 6BA **Telephone**: +44 (0) 7958036242  **Email**: [david.mccance@belfasttrust.hscni.net](mailto:david.mccance@belfasttrust.hscni.net) |
| **Public title** | **Pragmatic Lifestyle Pregnancy and Post Pregnancy Intervention for Overweight Women With Gestational Diabetes Mellitus (PAIGE2)** |
| **Scientific title** | **Pragmatic Lifestyle Pregnancy and Post Pregnancy Intervention for Overweight Women With Gestational Diabetes Mellitus (PAIGE2)** |
| **Countries of recruitment** | **Northern Ireland and the Republic of Ireland** |
| **Health condition(s) or problem(s) studied** | **Pregnancy and Post Pregnancy, Weight, Gestational Diabetes Mellitus** |
| **Intervention(s)** | Women in the control arm will receive usual antenatal care alone including access to an educational website. This resource is used within BHSCT clinics to educate women on the likely symptoms, causes, consequences, and management of GDM, including information on weight management and physical activity both before and after pregnancy as recommended by NICE guidelines*.  The experimental arm will receive access to the same educational website as the control group, but in addition will be enrolled in a lifestyle intervention comprising a one-hour educational programme delivered via telephone or video call during (32-36 weeks gestation). A fitness tracker and smartphone app will be offered to the intervention group at this point to help track their activity and daily step counts. In the early post-partum period, they will be offered a free three-month referral to a commercial weight management organization (Slimming World) scheme and intermittent contact will be made via texts and structured phone calls. Participants who are successful during the first three-month period of enrolment with a commercial weight management organization (gauged by attending at least 9 out of 12 sessions or demonstrated weight loss), will be offered a second funded three-month membership in order to maximize any benefits obtained during the program.  *At the end of the study, the control group will be offered referral to Slimming World (12 weeks duration) free of charge and will be given a copy of the educational resources from the one-hour educational session. |
| **Key inclusion and exclusion criteria** | **Inclusion criteria:**   1. Women aged 18 years of age or older. 2. Women with a booking BMI ≥ 25 kg/m2 at <14 weeks gestation. 3. GDM diagnosed in the current pregnancy. GDM will be defined by the WHO (2013) criteria (fasting plasma glucose (FPG) ≥ 5.1 mmol/l or 1h postprandial glucose ≥ 10.0 mmol/l or 2h plasma glucose ≥8.5 mmol/l). Women with a history of GDM directly preceding the current pregnancy, and who were given lifestyle advice and were performing self-monitoring of blood glucose from early pregnancy, will also be included if capillary glucose monitoring exceeds target values (fasting ≥ 5.3 mmol/l (95 mg/dl) or 1h postprandial plasma glucose ≥ 7.8 mmol/l (140 mg/dl)) thus negating the need for an OGTT. 4. Women will only continue to be included in the study if their fasting plasma glucose the morning after delivery or at 6 weeks postpartum is either normal or consistent with impaired fasting plasma glucose outside of pregnancy (i.e., <7 mmol/l). If the woman requests an earlier discharge that does not allow time for the immediate post-delivery FPG test, she will be offered an appointment to return to the maternity unit within 4-6 weeks of birth for retesting.   **Exclusion criteria**   1. Pregnancy in which an anomaly has been detected on the 20-week fetal anomaly scan. 2. History of diabetes outside of pregnancy or FPG ≥7 mmol/l the morning after delivery or at 6 weeks postpartum. Women with a FPG ≥7 mmol/l the morning after delivery or at 6 weeks postpartum will be referred to the local diabetes care team and excluded from the study. 3. History of heart, liver, or chronic renal disease. 4. Medications that adversely affect glucose tolerance (e.g., steroids). 5. Inability to participate in moderate physical activity outside of pregnancy. 6. Moderate/severe depressive illness or excess alcohol consumption. 7. Inability to understand adequately verbal explanations or written information in English, or special communication needs. 8. Women who are planning another pregnancy within the next 12 months following delivery. 9. Women who are intending to attend a CWMO program after pregnancy. 10. Exclusion criteria for early post-partum glucose testing will include massive postpartum hemorrhage, steroid treatment prior to delivery or patient refusal. |
| **Study type** | Interventional Allocation: randomized Intervention model: parallel assignment Masking: None Primary purpose: Health services research |
| **Date of first enrolment** | October 2020 |
| **Target sample size** | 340 women |
| **Recruitment status** | Completed |
| **Primary outcome(s)** | Weight at 12 months (taking into account any difference in weight at baseline between the intervention and control groups) |
| **Key secondary outcomes** | 1. Fasting glucose 2. Waist circumference 3. Step counts, change from baseline at 12 months (intervention group only) 4. Questionnaire data pertaining to physical activity, General Health and Well Being (including depression), Motivation to change and Risk Perception Survey for Developing Diabetes (RPS-DD)   **Other outcomes:**   1. Compliance: Slimming World records including attendance, number of visits and weight at first and last visits (intervention group only) 2. Blood Pressure: Blood pressure (automated, average of last 2 of 3 measurements taken in non-dominant arm after being seated for 5 minutes) 3. Follow up analysis of those women who have a further pregnancy within 12 months to document: BMI at booking, development of GDM and birth weight 4. Partner weekly step count (if applicable) |
