# Supplementary material for: A pragmatic lifestyle intervention for overweight and obese women with gestational diabetes mellitus (PAIGE2): A parallel arm, multicenter randomized controlled trial study protocol
Source: Front Clin Diabetes Healthc. 2023 Mar 24;4:1118509. doi: 10.3389/fcdhc.2023.1118509 (PMC10080069; doi:10.3389/fcdhc.2023.1118509)
Supplement: Supplementary file 2 [file Table_2.docx]

Supplementary Material 2

Standard Protocol Items: Recommendations for Intervention Trials (SPIRIT) Checklist

An-Wen Chan, Jennifer M. Tetzlaff, Douglas G. Altman, et al; SPIRIT 2013 Statement: Defining Standard Protocol Items for Clinical Trials. Ann Intern Med. 2013;158:200-207.
doi:10.7326/0003-4819-158-3-201302050-00583

| **Item** | **Item number and description** | **Page #** |
| --- | --- | --- |
| **Title** | Item 1: Descriptive title identifying the study design, population, interventions, and, if applicable, trial acronym. | 1 |
| **Registry** | Item 2a: Trial identifier and registry name. If not yet registered, name of intended registry. | 2 |
| **Data Set** | Item 2b: All items from the World Health Organization Trial Registration Data Set. | Supplementary Information 1 |
| **Protocol Version** | Item 3: Date and version identifier. | N/A |
| **Funding** | Item 4: Sources and types of financial, material, and other support. | 14 |
| **Contributorship** | Item 5a: Names, affiliations, and roles of protocol contributors. | 1, 13-14 |
| **Sponsor Contact Information** | Item 5b: Name and contact information for the trial sponsor. | 13 |
| **Sponsor And Funder** | Item 5c: Role of study sponsor and funders, if any, in study design; collection, management, analysis, and interpretation of data; writing of the report; and the decision to submit the report for publication, including whether they will have ultimate authority over any of these activities. | 14 |
| **Committees** | Item 5d: Composition, roles, and responsibilities of the coordinating centre, steering committee, endpoint adjudication committee, data management team, and other individuals or groups overseeing the trial, if applicable (see[Item 21a](https://www.spirit-statement.org/formal-committee/) for Data Monitoring Committee). | 13-14 |
| **Background And Rationale** | Item 6a: Description of research question and justification for undertaking the trial, including summary of relevant studies (published and unpublished) examining benefits and harms for each intervention. | 3-5 |
| **Choice Of Comparators** | Item 6b: Explanation for choice of comparators. | 9 |
| **Objectives** | Item 7: Specific objectives or hypotheses. | 5 |
| **Trial Design** | Item 8: Description of trial design including type of trial (e.g., parallel group, crossover, factorial, single group), allocation ratio, and framework (e.g., superiority, equivalence, non-inferiority, exploratory). | 6 |
| **Study Setting** | Item 9: Description of study settings (e.g., community clinic, academic hospital) and list of countries where data will be collected. Reference to where list of study sites can be obtained. | 6 |
| **Eligibility Criteria** | Item 10: Inclusion and exclusion criteria for participants. If applicable, eligibility criteria for study centers and individuals who will perform the interventions (e.g., surgeons, psychotherapists). | 7 |
| **Interventions** | Item 11a: Interventions for each group with sufficient detail to allow replication, including how and when they will be administered. | 8-9 |
| **Modifications** | Item 11b: Criteria for discontinuing or modifying allocated interventions for a given trial participant (e.g., drug dose change in response to harms, participant request, or improving/worsening disease). | N/A |
| **Adherence** | Item 11c: Strategies to improve adherence to intervention protocols, and any procedures for monitoring adherence (e.g., drug tablet return; laboratory tests). | N/A |
| **Concomitant Care** | Item 11d: Relevant concomitant care and interventions that are permitted or prohibited during the trial. | N/A |
| **Outcomes** | Item 12: Primary, secondary, and other outcomes, including the specific measurement variable (e.g., systolic blood pressure), analysis metric (e.g., change from baseline, final value, time to event), method of aggregation (e.g., median, proportion), and time point for each outcome. Explanation of the clinical relevance of chosen efficacy and harm outcomes is strongly recommended. | 10-12 |
| **Participant Timeline** | Item 13: Time schedule of enrolment, interventions (including any run-ins and washouts), assessments, and visits for participants. A schematic diagram is highly recommended (see Figure 1). | Figure 1 |
| **Sample Size** | Item 14: Estimated number of participants needed to achieve study objectives and how it was determined, including clinical and statistical assumptions supporting any sample size calculations. | 7 |
| **Recruitment** | Item 15: Strategies for achieving adequate participant enrolment to reach target sample size. | 6 |
| **Sequence Generation** | Item 16a: Method of generating the allocation sequence (e.g., computer-generated random numbers), and list of any factors for stratification. To reduce predictability of a random sequence, details of any planned restriction (e.g., blocking) should be provided in a separate document that is unavailable to those who enroll participants or assign interventions. | 6-7 |
| **Concealment Mechanism** | Item 16b: Mechanism of implementing the allocation sequence (e.g., central telephone; sequentially numbered, opaque, sealed envelopes), describing any steps to conceal the sequence until interventions are assigned. | 6-7 |
| **Implementation** | Item 16c: Who will generate the allocation sequence, who will enroll participants, and who will assign participants to interventions. | 6-7 |
| **Blinding (Masking)** | Item 17a: Who will be blinded after assignment to interventions (e.g., trial participants, care providers, outcome assessors, data analysts), and how. | 6-7 |
| **Emergency Unblinding** | Item 17b: If blinded, circumstances under which unblinding is permissible, and procedure for revealing a participant’s allocated intervention during the trial. | N/A |
| **Data Collection Methods** | Item 18a: Plans for assessment and collection of outcome, baseline, and other trial data, including any related processes to promote data quality (e.g., duplicate measurements, training of assessors) and a description of study instruments (e.g., questionnaires, laboratory tests) along with their reliability and validity, if known. Reference to where data collection forms can be found, if not in the protocol. | 9-12 |
| **Retention** | Item 18b: Plans to promote participant retention and complete follow-up, including list of any outcome data to be collected for participants who discontinue or deviate from intervention protocols. | 12 |
| **Data Management** | Item 19: Plans for data entry, coding, security, and storage, including any related processes to promote data quality (e.g., double data entry; range checks for data values). Reference to where details of data management procedures can be found, if not in the protocol. | 12 |
| **Statistical Methods – Outcomes** | Item 20a: Statistical methods for analyzing primary and secondary outcomes. Reference to where other details of the statistical analysis plan can be found, if not in the protocol. | 12-13 |
| **Additional Analyses** | Item 20b: Methods for any additional analyses (e.g., subgroup and adjusted analyses). | 12-13 |
| **Analysis Population And Missing Data** | Item 20c: Definition of analysis population relating to protocol non-adherence (e.g., as randomized analysis), and any statistical methods to handle missing data (e.g., multiple imputation). | 12-13 |
| **Formal Committee** | Item 21a: Composition of data monitoring committee (DMC); summary of its role and reporting structure; statement of whether it is independent from the sponsor and competing interests; and reference to where further details about its charter can be found, if not in the protocol. Alternatively, an explanation of why a DMC is not needed. | 12 |
| **Interim Analysis** | Item 21b: Description of any interim analyses and stopping guidelines, including who will have access to these interim results and make the final decision to terminate the trial. | N/A |
| **Harms** | Item 22: Plans for collecting, assessing, reporting, and managing solicited and spontaneously reported adverse events and other unintended effects of trial interventions or trial conduct. | N/A |
| **Auditing** | Item 23: Frequency and procedures for auditing trial conduct, if any, and whether the process will be independent from investigators and the sponsor. | N/A |
| **Research Ethics Approval** | Item 24: Plans for seeking research ethics committee/institutional review board (REC/IRB) approval. | 13 |
| **Protocol Amendments** | Item 25: Plans for communicating important protocol modifications (e.g., changes to eligibility criteria, outcomes, analyses) to relevant parties (e.g., investigators, REC/IRBs, trial participants, trial registries, journals, regulators). | N/A |
| **Consent Or Assent** | Item 26a: Who will obtain informed consent or assent from potential trial participants or authorized surrogates, and how (see Item 32). | 6 |
| **Ancillary Studies** | Item 26b: Additional consent provisions for collection and use of participant data and biological specimens in ancillary studies, if applicable. | 12 |
| **Confidentiality** | Item 27: How personal information about potential and enrolled participants will be collected, shared, and maintained to protect confidentiality before, during, and after the trial. | 12 |
| **Declaration Of Interests** | Item 28: Financial and other competing interests for principal investigators for the overall trial and each study site. | 14 |
| **Access To Data** | Item 29: Statement of who will have access to the final trial dataset, and disclosure of contractual agreements that limit such access for investigators. | 14 |
| **Ancillary And Post-Trial Care** | Item 30: Provisions, if any, for ancillary and post-trial care, and for compensation to those who suffer harm from trial participation. | N/A |
| **Trial Results** | Item 31a: Plans for investigators and sponsor to communicate trial results to participants, healthcare professionals, the public, and other relevant groups (e.g., via publication, reporting in results databases, or other data sharing arrangements), including any publication restrictions. | 13 |
| **Authorship** | Item 31b: Authorship eligibility guidelines and any intended use of professional writers. | N/A |
| **Reproducible Research** | Item 31c: Plans, if any, for granting public access to the full protocol, participant-level dataset, and statistical code. | N/A |
| **Informed Consent Materials** | Item 32: Model consent form and other related documentation given to participants and authorized surrogates. | N/A |
| **Biological Specimens** | Item 33: Plans for collection, laboratory evaluation, and storage of biological specimens for genetic or molecular analysis in the current trial and for future use in ancillary studies, if applicable. | N/A |
